# Associations of self-reported physical activity and depression in 10,000 Irish adults across harmonised datasets: a DEDIPAC-study

**DOI:** 10.1186/s12889-018-5702-4

**Published:** 2018-07-01

**Authors:** Cillian P. Mc Dowell, Angela Carlin, Laura Capranica, Christina Dillon, Janas M. Harrington, Jeroen Lakerveld, Anne Loyen, Fiona Chun Man Ling, Johannes Brug, Ciaran MacDonncha, Matthew P. Herring

**Affiliations:** 1 Department of Physical Education and Sport Sciences, University of Limerick, Limerick, Ireland; 2 Centre for Exercise Medicine, Physical Activity and Health, School of Sport, Ulster University, Belfast, Northern Ireland; 3 Department of Movement, Human and Health Sciences, University of Rome Foro Italico, Rome, Italy; 4 National Suicide Research Foundation, University College Cork, Cork, Ireland; 5 School of Public Health, University College Cork, Cork, Ireland; 6 Department of Epidemiology and Biostatistics, Amsterdam Public Health research institute, VU University Medical Center, Amsterdam, The Netherlands; 7 Department of Social and Occupational Health, Amsterdam Public Health research institute, VU University Medical Center, Amsterdam, the Netherlands; 8 Institute of Sport, Exercise and Active Living, Victoria University, Melbourne, Australia; 9 Department of Psychology, Faculty of Science & Technology, Bournemouth University, Bournemouth, UK; 10 Amsterdam School for Communication Research, University of Amsterdam, Amsterdam, the Netherlands; 11 Health Research Institute, University of Limerick, Limerick, Ireland

**Keywords:** Physical activity, Mental health, Elderly, Ireland, Cross-sectional

## Abstract

**Background:**

Depression is a prevalent, debilitating, and often recurrent mood disorder for which successful first-line treatments remains limited. The purpose of this study was to investigate the cross-sectional associations between self-reported physical activity (PA) and depressive symptoms and status among Irish adults, using two existing datasets, The Irish Longitudinal Study on Ageing (TILDA) and The Mitchelstown Cohort Study.

**Methods:**

The two selected databases were pooled (*n* = 10,122), and relevant variables were harmonized. PA was measured using the short form International Physical Activity Questionnaire. Depressive symptoms were measured by the Center for Epidemiologic Studies Depression (CES-D) questionnaire. Participants were classified as meeting World Health Organization moderate-to-vigorous PA (MVPA) guidelines or not, and divided into tertiles based on weekly minutes of MVPA. A CES-D score of ≥16 indicated elevated depressive symptoms. Data collection were conducted in 2010–2011.

**Results:**

Significantly higher depressive symptoms were reported by females (7.11 ± 7.87) than males (5.74 ± 6.86; *p* < 0.001). Following adjustment for age, sex, BMI, and dataset, meeting the PA guidelines was associated with 44.7% (95%CI: 35.0 to 52.9; *p* < 0.001) lower odds of elevated depressive symptoms. Compared to the low PA tertile, the middle and high PA tertiles were associated with 25.2% (95%CI: 8.7 to 38.6; *p* < 0.01) and 50.8% (95%CI: 40.7 to 59.2; *p* < 0.001) lower odds of elevated depressive symptoms, respectively.

**Conclusion:**

Meeting the PA guidelines is associated with lower odds of elevated depressive symptoms, and increased volumes of MVPA are associated with lower odds of elevated depressive symptoms.

**Electronic supplementary material:**

The online version of this article (10.1186/s12889-018-5702-4) contains supplementary material, which is available to authorized users.

## Background

Depression is a prevalent, debilitating, and often recurrent mood disorder [1, 2] that affects over 300 million people globally [1] and is the second leading cause of global disability [3]. The financial burden associated with depression continues to grow, due largely to increasing treatment costs and wider societal costs of lost productivity [4]. Depression is the mental disorder with the highest cost implications in Europe, accounting for over €90 billion in direct and indirect cost across Europe [4].

Successful treatment of depression with traditional first-line treatments, such as psychotherapy and pharmacotherapy, remains limited [5]. Exercise has been show to be a viable treatment for depression and depressive symptoms [6, 7]; however, evidence indicates that approximately 63% of people with mental health problems, including depression, do not seek specialist treatment and only half of those who do appear to improve [8]. Thus, there is a continued need to identify and investigate cost-effective prevention and treatment strategies that are effective and more accessible.

The salutary benefits of regular physical activity (PA) for mental health are well-established [9] and without the physiologic side effects and costs associated with antidepressants and psychotherapy [10]. In the context of depression, evidence supports: (i) significantly lower PA levels among adults with depression compared to adults without depression [11, 12]; (ii) inverse associations between PA and onset of depression [13, 14]; and (iii) that habitual PA may moderate treatment response [15]. However, many studies have not used validated instruments to capture important features of PA such as type, frequency, intensity, and duration, and the use of inconsistent self-report measures of PA between studies has limited the ability to examine dose–response relationships between PA and depression [13]. Despite this, large-scale studies exist that have measured PA and depression, though this research question often was not the primary focus of the original research [16]. Furthermore, in studies that did address PA as potential correlate of depression, different methodological approaches have been adopted, limiting the possibility to generalize the findings [13].

The cost of gathering such data is significant and research funders often emphasize maximising the potential of existing data resources through data sharing and secondary analysis [17–19]. The research and knowledge potential of existing datasets can be further exploited through dataset pooling and subsequent harmonisation of variables [17, 20–23]. Reported benefits of pooling include increased statistical power, potential for subgroup analysis and comparative research, increased exposure heterogeneity and generalisability, and increased cost-efficiency of research programmes [17, 24–26].

Thus, the aim of the current investigation was to harmonise and integrate two existing datasets in order to increase statistical power and generalisability in investigating and quantifying the association between meeting PA guidelines and elevated depressive symptoms, and to explore whether larger associations are observed with increased PA, in a large cross-sectional population sample of Irish adults. It is hypothesised that meeting PA guidelines will be associated with lower odds of elevated depressive symptoms, with lower odds of elevated depressive symptoms observed with greater volumes of PA.

## Methods

This study used Strengthening the Reporting of Observational Studies in Epidemiology (STROBE) recommendations to guide reporting [27].

### Selection of participating studies

The platform for the current research was the DEterminants of DIet and Physical ACtivity Knowledge Hub (DEDIPAC-KH), a pan-European joint programme initiative active from 2013 to 2016. The focus of DEDIPAC was to better understand how individual and contextual factors influence diet, PA, and sedentary behaviour [28, 29]. Within DEDIPAC, a compendium of relevant and accessible datasets regarding the listed health risk behaviours and their determinants was developed [30]. Datasets for inclusion in the compendium were identified by: i) a search of the CORDIS project platform, which is the European Commission’s primary public repository and portal to disseminate information on all EU-funded research projects and their results, ii) an examination of existing recent literature reviews and noting the nature of datasets used, and iii) recommendation from the DEDIPAC network and expert consultation. The compendium provides information on the study, the study population, the determinants and health behaviours measured and level of accessibility. The compendium is accessible through https://www.dedipac.eu.

On examination of the compendium, 23 datasets were noted that measured both PA behaviour and a psychological determinant of some nature (e.g. motivation, perceived competence, anxiety, depression etc.). An evaluation of the available data dictionaries which were either publicly available or secured directly from the targeted dataset owners revealed five relevant datasets (three Irish, one Dutch, and one multi-nation) which had potential for harmonisation (i.e., PA and depression measured using a same or similar methodology). Target variables (including relevant covariates) were agreed and listed by three authors (CMcD, CMacD, MPH). The dataset owners were subsequently approached for access to the specific target variables. Of these, all agreed to participate; however, the multi-national study was inaccessible within the timeframe of the study. Therefore, the Dutch study was also excluded due to its smaller sample and nationality that differed from the remaining three studies. Another study (the Cork Children’s Lifestyle Survey) was subsequently excluded as it assessed lifetime diagnosis of depression rather than current depressive symptoms. Appropriate data access agreements were signed as required. Only cross-sectional data for datasets were available. Thus, two datasets, The Irish Longitudinal Study on Ageing (TILDA) and The Mitchelstown Cohort Study together consisting of 10,122 Irish adults, were harmonised for the present study.

### Participating datasets

The Irish Longitudinal Study on Ageing (TILDA) is a large (*n* = 8329) prospective cohort study that assesses the social, economic, and health circumstances of community dwelling adults aged ≥50 years and their partners of any age in Ireland [31]. For the current study, the first wave of data collected in 2011 was used. PA was measured using the short International Physical Activity Questionnaire (IPAQ-SF) [32], in which respondents are asked to report the number of days and the duration of the vigorous, moderate, and walking activities they undertook during the last seven days. Depression was measured with the Center for Epidemiologic Studies Depression (CES-D) questionnaire [33].

The Mitchelstown Cohort Study provided data (*n* = 1804) from the second phase of the Cork and Kerry Diabetes and Heart Disease Study [34], designed to assess diabetes and heart disease among 55–74 year-old adults. Data collection began in 2010. PA was measured with the IPAQ-SF while depression was measured using the CES-D.

### Harmonisation process

A target list of relevant variables that may influence the association between PA and depression, based on a logical, theoretical, or prior empirical relation with PA and/or depression, and that had potential for data harmonisation (i.e., same or similar measure) was generated from the two included datasets. Appropriate variable harmonisation methods for PA, depression, and participant age, sex, and body mass index (BMI) were agreed by a six-person expert consensus group from within DEDIPAC (Table 1). These variables were then harmonised to form a large PA and mental health integrated dataset.

**Table 1:** Information on data harmonisation across datasets

Concept measured	TILDA	Mitchelstown Cohort Study	Harmonisation method
PA	IPAQ – Self-reported weekly minutes of moderate and vigorous PA	IPAQ – Self-reported weekly minutes of moderate and vigorous PA	Weekly minutes of moderate and vigorous PA were summed. Participants were categorised as meeting/not meeting PA guidelines and divided into tertiles of weekly minutes of MVPA within each study and across the integrated dataset
Depression	CES-D (continuous score)	CES-D (continuous score)	Participants with CES-D scores of ≥16 were classified as at-risk for depression
Age	Age reported for 50–79 years (ages < 50 reported as 49; ages > 79 were reported as 80)	Age (5 year categories)	Divided into 10 year categories
Sex	Female and male	Female and male	Females and males grouped
BMI	BMI (categorical) calculated from nurse measured height weight	BMI (continuous) calculated from measured height weight	Split into underweight, normal weight, overweight, and obese using standard cut-offs

*BMI* Body mass index, *CES-D* Center for Epidemiological Studies Depression, *IPAQ* International physical activity questionnaire, *PA* Physical activity; *TILDA* The Irish longitudinal study on ageing

### Data analysis

PA data were processed according to the IPAQ guidelines for data processing and analysis [35], and respondents were categorised as meeting or not meeting World Health Organization PA guidelines (i.e., ≥150 min.week^− 1^ of MVPA, ≥75 min.week^− 1^ of vigorous PA, or ≥ 500 MET.min.week^− 1^) [36]. As the PA data were positively skewed, within individual datasets and across the integrated dataset participants were divided into tertiles based on weekly minutes of MVPA (i.e., 0 min.week^− 1^, 10–295 min.week^− 1^, and ≥ 300 min.week^− 1^). CES-D scores range from 0 to 60, and individuals at risk for clinical depression were indicated by scores ≥16 [33]; this cut-off score has demonstrated 100% sensitivity and 87.6% specificity among adults [37]. Respondents above this cut-off score are referred to as having elevated depressive symptoms throughout the current manuscript as they are at increased risk of depression; however, meeting the cut-off score does not necessarily indicate the presence of clinically diagnosed depression. BMI was categorized as underweight, normal weight, overweight, and obese using established thresholds of < 18.5 kg.m^− 2^, 18.5–24.99 kg.m^− 2^, 25–29.99 kg.m^− 2^, and ≥ 30 kg.m^− 2^, respectively [38]. Statistical analyses were conducted using SPSS Version 22.0 (Armonk, NY: IBM Corp.).

#### Within dataset analyses

For descriptive purposes, Chi-square tests examined differences in proportions of elevated depressive symptom status, ten-year age categories, sex, BMI categories, and meeting PA guidelines between datasets. For significant Chi-square tests, *Z* tests were calculated for column proportions for each row in the Chi-square contingency table and adjusted using a Bonferroni correction to provide further detail regarding PA and depression across the different levels of covariates [39]. Within each dataset, binomial logistic regression quantified crude and adjusted associations between PA and elevated depressive symptoms. Covariates in adjusted models were age, sex, and BMI.

#### Integrated dataset analysis

Chi-square tests examined differences in elevated depressive symptom status, age categories, sex, and BMI categories, between individuals meeting and not meeting PA guidelines. Binomial logistic regression quantified crude and adjusted associations between PA and elevated depressive symptom status. Covariates in adjusted models were age, sex, BMI, and dataset. Likelihood ratio tests examined covariate significance. One-way ANOVAs followed by Bonferroni-corrected *post-hoc* tests quantified differences in depressive symptoms between those meeting and not meeting PA guidelines, PA tertiles, and sexes. Hedges’ *g* effect sizes and associated 95% confidence intervals (95%CI) were calculated to quantify the magnitude of differences in depressive symptom scores between meeting PA guidelines, PA tertiles, and sexes [40]. Effect sizes of 0.2, 0.5, and 0.8 can been used as cut-offs for small, medium, and large effects, respectively [41].

## Results

A total of 10,133 and 7809 respondents were included in crude and adjusted analyses, respectively. The overall prevalence of elevated depressive symptoms was 10.9%. Females were more likely to report elevated depressive symptoms (χ^2^ (1, *N* = 10,133) = 54.49, *p* < 0.001) than males (12.9% vs 8.3%), and reported significantly higher depressive symptoms (7.11 ± 7.87) than men (5.74 ± 6.86; F_(1,10,131)_ = 84.59, *p* < 0.001; *g* = 0.18; 95%CI: 0.15 to 0.22).

### Participant characteristics within datasets

Harmonised data characteristics are presented in Table 2. Elevated depressive symptom status (χ^2^ (1, N = 10,133) = 65.79, *p* < 0.001), meeting PA guidelines (χ^2^ (1, N = 10,133) = 156.61, *p* < 0.001), sex (χ^2^ (2, N = 10,133) = 11.57, *p* < 0.001), and age (χ^2^ (4, N = 10,122) = 664.55, *p* < 0.001 were significantly different between studies. Results from follow-up *Z* tests are shown in Table 2. Briefly, a significantly greater proportion of Mitchelstown participants reported elevated depressive symptoms, did not meet PA guidelines, and were aged 50–59 years and male (all *p* < 0.05).

**Table 2:** Study characteristics

	TILDA	Mitchelstown
MVPA (minutes (median IQR))	90.00 (600.00)	0.00 (180.00)
PA (n (%))		
Not Meeting PA guidelines	4471 (53.8)	1259 (69.0)
Meeting PA guidelines	3858 (46.3)	545 (30.2)
Depression status (n (%))		
Not Depressed	7522 (90.3)	1511 (83.8)
Depressed	807 (9.7)	293 (16.2)
Age (n (%))		
< 50	327 (3.9)_a_	2 (0.1)_b_
50–59	3210 (38.6)_a_	973 (53.9)_b_
60–69	2540 (30.5)_a_	801 (44.4)_b_
70–79	1636 (19.7)_a_	28 (1.6)_b_
80+	605 (7.3)_a_	0 (0.0)_b_
Sex (n (%))		
Male	3706 (44.5)_a_	882 (48.9)_b_
Female	4623 (55.5)_a_	922 (51.1)_b_
BMI (n (%))		
Underweight	32 (0.5)	7 (0.4)
Normal	1353 (22.5)	401 (22.1)
Overweight	2576 (42.8)	821 (45.3)
Obese	2056 (34.2)	584 (32.2)

Different subscript letters indicate a subset of each category whose column proportions differ statistically significantly at the .05 level

*BMI* Body mass index, *MVPA* Moderate-to-vigorous physical activity, *TILDA* The Irish longitudinal study on ageing

### Within dataset analyses

Table 3 presents crude and adjusted odds ratios for the associations between meeting PA guidelines and PA tertiles, and elevated depressive symptoms within each dataset. Significant crude associations remained robust following adjustment for age, sex, and BMI. Meeting PA guidelines was associated with 49.8% (95% CI: 39.2 to 58.5; *p* < 0.001) and 27.8% (95% CI: 2.8 to 46.4; *p* < 0.05) lower odds of elevated depressive symptoms in TILDA and Mitchelstown, respectively.

**Table 3:** Odds ratios (OR) and 95% confidence intervals (CI) derived from binominal logistic regression analyses as indicators of associations between physical activity (PA) and depressive symptoms

	TILDA OR (95%CI)	Mitchelstown OR (95%CI)	Integrated Dataset OR (95%CI)
Crude			
PA Guidelines			
Not Meeting PA guidelines	REF	REF	REF
Meeting PA guidelines	0.509 (0.436 to 0.594)	0.685 (0.512 to 0.915)	0.514 (0.449 to 0.589)
Adjusted			
PA Guidelines			
Not Meeting PA guidelines	REF	REF	REF
Meeting PA guidelines	0.502 (0.415 to 0.608)	0.722 (0.536 to 0.972)	0.557 (0.474 to 0.655)
Crude			
PA Tertiles			
Low PA Tertile	REF	REF	REF
Middle PA Tertile	0.681 (0.560 to 0.829)	0.704 (0.492 to 1.008)	0.663 (0.558 to 0.787)
High PA Tertile	0.469 (0.393 to 0.559)	0.561 (0.393 to 0.803)	0.455 (0.389 to 0.532)
Adjusted			
PA Tertiles			
Low PA Tertile	REF	REF	REF
Middle PA Tertile	0.736 (0.585 to 0.926)	0.698 (0.485 to 1.005)	0.736 (0.606 to 0.893)
High PA Tertile	0.454 (0.364 to 0.567)	0.615 (0.426 to 0.888)	0.489 (0.404 to 0.591)

Similar to crude associations, following adjustment for age, sex, and BMI, the middle PA tertile was associated with 26.4% (95% CI: 7.4 to 41.5; *p* ≤ 0.001) and 30.2% (95% CI: -0.5 to 51.5; *p* > 0.05) lower odds of elevated depressive symptoms in TILDA and Mitchelstown, respectively; the high PA tertile was associated with 54.6% (95% CI: 43.3 to 63.6; *p* < 0.001) and 38.5% (95% CI: 11.2 to 57.4; *p* < 0.01) lower odds of elevated depressive symptoms in TILDA and Mitchelstown, respectively.

### Participant characteristics across integrated dataset

Participant characteristics by PA guideline adherence in the integrated dataset are presented in Table 4. Elevated depressive symptom status (χ^2^ (1, *N* = 10,133) = 94.60, *p* < 0.001), sex (χ^2^ (1, N = 10,133) = 243.30, *p* < 0.001), age (χ^2^ (4, N = 10,122) = 168.63, *p* < 0.001), and BMI (χ^2^ (3, *N* = 7818) = 22.03, *p* < 0.001) significantly differed between meeting and not meeting PA guidelines. Briefly, people not meeting PA guidelines were significantly more likely to report elevated depressive symptoms and be female, aged 70–79 or ≥ 80 years, and obese (all *p* < 0.05).

**Table 4:** Participant characteristics

	Meeting PA Guidelines (n (%))	Not Meeting PA Guidelines (n (%))
Depression status		
Depressed	327 (7.4)	773 (13.5)
Not depressed	4076 (92.6)	4957 (86.5)
Age (years)		
< 50	175 (4.0)_a_	154 (2.7)_b_
50–59	1987 (45.2)_a_	2196 (38.4)_b_
60–69	1482 (33.7)_a_	1859 (32.5)_a_
70–79	609 (13.9)_a_	1055 (18.4)_b_
80+	144 (3.3)_a_	461 (8.1)_b_
Sex		
Male	2381 (54.1)	2207 (38.5)
Female	2022 (45.9)	3523 (61.5)
BMI		
Underweight	15 (0.4)_a_	24 (0.6)_a_
Normal	787 (22.7)_a_	966 (22.2)_a_
Overweight	1589 (45.8)_a_	1802 (41.4)_b_
Obese	1078 (31.1)_a_	1557 (35.8)_b_

Different subscript letters indicate a subset of each category whose column proportions differ statistically significantly at the .05 level

*BMI* Body mass index, *PA* Participant characteristics

### Integrated dataset analyses

Table 3 presents crude and adjusted odds ratios for the associations between meeting PA guidelines and PA tertiles, and elevated depressive symptoms across the integrated dataset. Odds ratios for covariates in adjusted analyses are reported in Additional file 1: Table S1. Significant crude associations remained robust following adjustment for age, sex, BMI, and dataset. Compared to not meeting PA guidelines, meeting the guidelines was associated with 44.3% (95% CI: 34.5 to 52.6; *p* < 0.001) lower odds of elevated depressive symptoms. Age, sex, and BMI were significant covariates (all *p* < 0.005).

Significant crude associations remained robust following adjustment for age, sex, BMI, and dataset. The middle and high PA tertiles were associated with 26.4% (95% CI: 10.7 to 39.4; *p* < 0.01) and 51.1% (95% CI: 40.9 to 59.6; *p* < 0.001) lower odds of elevated depressive symptoms, respectively. Age, sex, and BMI were significant covariates (all *p* < 0.05).

Depressive symptoms were significantly higher among people not meeting MVPA guidelines (7.43 ± 7.95) than those meeting PA guidelines (5.30 ± 6.59; F_(1,10,131)_ = 200.71, *p* < 0.001; *g* = 0.28; 95%CI: 0.25 to 0.32). Depressive symptoms significantly differed by PA tertile (*p* < 0.001). *Post-hoc* tests showed significantly lower depressive symptoms for the high PA tertile (5.05 ± 6.53) compared to the low (7.60 ± 8.03, *p* < 0.001; *g* = 0.34 95%CI: 0.30 to 0.39) and middle (6.16 ± 6.99, *p* < 0.001; *g* = 0.17; 95%CI: 0.11 to 0.22) PA tertiles. The middle PA tertile also reported significantly lower depressive symptoms than the low PA tertile (*p* < 0.001; *g* = 0.19; 95%CI: 0.13 to 0.24).

## Discussion

Using secondary analyses of pooled data from large-scale Irish studies, this work contributes to deepen the knowledge on the association between meeting PA guidelines and elevated depressive symptoms, and a potential dose-response in the PA-depression relationship, in a European country. A previous umbrella systematic review of psychological determinants of PA across the life course highlighted that the negative association between PA and depression/depressive symptoms’ presents limited and no conclusive evidence [42]. The present study supports associations between meeting PA guidelines and lower odds of depression, and a dose response in the PA-depression relationship.

Approximately 10.9% of the current sample reported elevated depressive symptoms, supporting previous findings in worldwide [2] and Irish [43] samples. Females were significantly more likely to report higher depressive symptoms (*g* = 0.18), which is consistent with previous evidence [44]. Adjusted analyses, both within and across datasets, support the association between not meeting MVPA guidelines and elevated depressive symptoms, consistent with previous findings regarding the beneficial effects of meeting MVPA guidelines on depressive symptoms [13]. The 44% lower odds of reporting elevated depressive symptoms among people meeting MVPA guidelines is consistent with previous findings [9, 45].

A potential dose–response relationship was observed in the present study. Compared to the low PA tertile, the middle and high PA tertiles were associated with 26 and 51% lower odds of elevated depressive symptoms, respectively, such that participation in the highest volume of MVPA appeared to confer more benefits than a more moderate volume. Previous findings are limited and contradictory, and a clear dose–response relationship between PA and depression has not been readily apparent [13, 15]. Research by Dunn et al. (2005) [46] observed similar findings to ours in that significantly lower post-intervention depression scores among adults with mild to moderate major depressive disorder whose total energy expenditure was 17.5 kcal/kg/week for 12-weeks compared to those whose total energy expenditure was 7.0 kcal/kg/week [46]. Further, those who routinely engage in higher levels of PA have been shown to respond better to depression treatment than those who are less active [15]. Collectively, these findings support the need to engage in at least a moderate level of PA, with the potential for increased benefits with increased PA.

Although not examined in the present study, there is also evidence to suggest that compared to engaging in no PA, engaging in volumes of MVPA less than recommended levels [13], and increasing levels of light intensity PA [47], may also protect against depression. This may have important public health implications as, although the current study strongly supports the benefits of meeting PA guidelines, the wider research appears to indicate that any amount of PA is better than none.

The iterative steps for effective retrospective data harmonisation published by Fortier and colleagues (2017) [24] underpinned the harmonisation process within this study (e.g. definition of question, detailed characteristics regarding potential datasets, expert input, valid data input, etc.). In concordance with an effective data harmonisation process, the dataset owners were involved from an early stage and access to detailed information on the data collection procedures adopted by the two studies included was available. The implementation of such methodological rigour optimised the quality of the harmonisation process and, thereby the quality and scientific utility of the final database generated.

### Strengths and limitations

The present study benefits from a large sample size, a PA measure that measured important components of PA, such as frequency, duration, and intensity of PA, direct examination of meeting PA guidelines, and following rigorous harmonisation procedures. However, several limitations exist. There is the possibility of differences in sampling methodology across studies, while some differences between studies can be impossible to reconcile. For example, including other lifestyle and health behaviours, socio-economic status, and social support as covariates may influence the association between PA level and depression, yet differences in how they were measured made it impossible to include it as a covariate in the present analyses. Because of this, the field will benefit from a more standardised set of measurements across studies. Over-reporting of PA is frequently encountered in epidemiological research partly due to influences such as social desirability and recall bias [48] and may have led to respondents being misclassified as meeting PA guidelines in the present study; however, there is no evidence to suggest that reporting error should differ according to mental health status. Finally, due to the cross-sectional design of this study, causation cannot be deduced. Nonetheless, the current findings are to the authors’ knowledge the first to provide support for the benefits of PA for depression, with indications of a dose-response relation, among a large, Irish sample.

## Conclusions

Inverse associations between meeting PA guidelines and odds of reporting elevated depressive symptoms were observed, with stronger associations observed with increased volumes of MVPA. Future prospective cohort studies should examine this potential dose-response relationship and focus on potential moderators and mediators of the association between PA and depression in large, European-based samples. Further, more large-scale prospective studies in large European-based samples using objective measure of PA are required to establish cause and effect and to further elucidate the role of potential moderators and mediators of the PA-depression relationship.

## Additional file

Additional file 1: **Table S1.** Odds ratios (OR) and 95% confidence intervals (CI) derived from binominal logistic regression analyses as indicators of association between physical activity (PA) and covariates and depressive symptoms. (DOCX 17 kb)

## Abbreviations

ANOVA: Analysis of Variance
BMI: Body Mass Index
CES-D: Center for Epidemiologic Studies Depression
DEDIPAC-KH: DEterminants of DIet and Physical ACtivity Knowledge Hub
IPAQ-SF: International Physical Activity Questionnaire – Short Form
kg: kilogram
m: meter
min: minutes
MVPA: Moderate-to-Vigorous Physical Activity
PA: Physical Activity
TILDA: The Irish Longitudinal Study on Ageing
