# Supplementary material for: Associations of self-reported physical activity and depression in 10,000 Irish adults across harmonised datasets: a DEDIPAC-study
Source: BMC Public Health. 2018 Jul 1;18:779. doi: 10.1186/s12889-018-5702-4 (PMC6026508; doi:10.1186/s12889-018-5702-4)
Supplement: Supplementary file 1 — Table S1. Odds ratios (OR) and 95% confidence intervals (CI) derived from binominal logistic regression analyses as indicators of association between physical activity (PA) and covariates and depressive symptoms. (DOCX 17 kb) [file 12889_2018_5702_MOESM1_ESM.docx]

**Table S1**. Odds ratios (OR) and 95% confidence intervals (CI) derived from binominal logistic regression analyses as indicators of association between physical activity (PA) and covariates and depressive symptoms

|  | **Integrated Dataset**  **OR (95%CI)** |
| --- | --- |
| **Model 1** |  |
| **PA Guidelines** |  |
| Not Meeting PA guidelines | **REF** |
| Meeting PA guidelines | 0.557 (0.474 to 0.655) |
| **Age (years)** |  |
| 80+ | **REF** |
| 70-79 | 1.887 (1.107 to 3.215) |
| 60-69 | 2.028 (1.217 to 3.378) |
| 50-59 | 2.529 (1.525 to 4.192) |
| <50 | 2.134 (1.120 to 4.067) |
| **Sex** |  |
| Male | **REF** |
| Female | 1.537 (1.315 to 1.796) |
| **BMI** |  |
| Normal | **REF** |
| Underweight | 1.161 (0.443 to 3.041) |
| Overweight | 0.935 (0.770 to 1.136) |
| Obese | 1.268 (1.042 to 1.543) |
| **Model 2** |  |
| **PA Tertiles** |  |
| Low | **REF** |
| Middle | 0.736 (0.606 to 0.893) |
| High | 0.489 (0.404 to 0.591) |
| **Age (years)** |  |
| 80+ | **REF** |
| 70-79 | 1.906 (1.118 to 3.250) |
| 60-69 | 2.058 (1.235 to 3.430) |
| 50-59 | 2.578 (1.554 to 4.275) |
| <50 | 2.165 (1.135 to 4.127) |
| **Sex** |  |
| Male | **REF** |
| Female | 1.519 (1.300 to 1.775) |
| **BMI** |  |
| Normal | **REF** |
| Underweight | 1.119 (0.427 to 2.931) |
| Overweight | 0.931 (0.767 to 1.131) |
| Obese | 1.265 (1.040 to 1.539) |
| Abbreviations: BMI:Body mass index; REF=Reference category; | |
